# Missense Mutation of Brain Derived Neurotrophic Factor (*BDNF*) Alters Neurocognitive Performance in Patients with Mild Traumatic Brain Injury: A Longitudinal Study

**DOI:** 10.1371/journal.pone.0158838

**Published:** 2016-07-20

**Authors:** Vairavan Narayanan, Vigneswaran Veeramuthu, Azlina Ahmad-Annuar, Norlisah Ramli, Vicknes Waran, Karuthan Chinna, Mark William Bondi, Lisa Delano-Wood, Dharmendra Ganesan

**Affiliations:** 1 Division of Neurosurgery, Department of Surgery, Faculty of Medicine, University of Malaya, Kuala Lumpur, Wilayah Persekutuan, Malaysia; 2 Department of Molecular Medicine, Faculty of Medicine, University of Malaya, 50603 Kuala Lumpur, Wilayah Persekutuan, Malaysia; 3 University Malaya Research Imaging Centre, University of Malaya, Kuala Lumpur, Wilayah Persekutuan, Malaysia; 4 Julius Centre University Malaya, Department of Social and Preventive Medicine, University of Malaya, Kuala Lumpur, Malaysia; 5 VA San Diego Healthcare System, San Diego, California, United States of America; 6 University of California San Diego, Department of Psychiatry, San Diego, California, United States of America; Texas Tech University Health Science Centers, UNITED STATES

## Abstract

The predictability of neurocognitive outcomes in patients with traumatic brain injury is not straightforward. The extent and nature of recovery in patients with mild traumatic brain injury (mTBI) are usually heterogeneous and not substantially explained by the commonly known demographic and injury-related prognostic factors despite having sustained similar injuries or injury severity. Hence, this study evaluated the effects and association of the Brain Derived Neurotrophic Factor (*BDNF)* missense mutations in relation to neurocognitive performance among patients with mTBI. 48 patients with mTBI were prospectively recruited and MRI scans of the brain were performed within an average 10.1 (SD 4.2) hours post trauma with assessment of their neuropsychological performance post full Glasgow Coma Scale (GCS) recovery. Neurocognitive assessments were repeated again at 6 months follow-up. The paired t-test, Cohen’s d effect size and repeated measure ANOVA were performed to delineate statistically significant differences between the groups [wildtype G allele (Val homozygotes) vs. minor A allele (Met carriers)] and their neuropsychological performance across the time point (T_1_ = baseline/ admission vs. T_2_ = 6^th^ month follow-up). Minor A allele carriers in this study generally performed more poorly on neuropsychological testing in comparison wildtype G allele group at both time points. Significant mean differences were observed among the wildtype group in the domains of memory (*M* = -11.44, SD = 10.0, p = .01, *d* = 1.22), executive function (*M* = -11.56, SD = 11.7, p = .02, *d* = 1.05) and overall performance (*M* = -6.89 SD = 5.3, p = .00, *d* = 1.39), while the minor A allele carriers showed significant mean differences in the domains of attention (*M* = -11.0, SD = 13.1, p = .00, *d* = .86) and overall cognitive performance (*M* = -5.25, SD = 8.1, p = .01, *d* = .66).The minor A allele carriers in comparison to the wildtype G allele group, showed considerably lower scores at admission and remained impaired in most domains across the timepoints, although delayed signs of recovery were noted to be significant in the domains attention and overall cognition. In conclusion, the current study has demonstrated the role of the BDNF rs6265 Val66Met polymorphism in influencing specific neurocognitive outcomes in patients with mTBI. Findings were more detrimentally profound among Met allele carriers.

## Introduction

Mild traumatic brain injury (mTBI) due to road traffic accident (RTA) is one of the most common forms of head injury, afflicting millions of people worldwide [1–3]. The complex pathophysiology of mTBI and the biochemical responses that occurs thereafter frequently results in cognitive, affective or behavioral deficits [4–6]. A wide variety of complaints and symptoms have been reported [7–11]. The predictability of these deficits are however not straightforward [12]. Crawford et al (2002) and Pruthi et al (2010) in their respective studies noted that the extent and nature of recovery in patients with mTBI are usually heterogeneous and not substantially explained by the commonly known demographic and injury-related prognostic factors [5, 13], despite having sustained similar injuries or injury severity [12, 14–15].

While there are many factors that may contribute to the outcome variability observed in mTBI, reliable genetic or imaging prognostic markers are sparse. In recent years, the expression and modulation of neurotropic genes, both normal and mutated, have been postulated as potential prognostic markers [16]. A wide range of aberrant genes including apolipoprotein E, dopamine β hydroxylase (DBH), cathecol-O-methyltransferase (COMT), calcium channel subunit gene (CACNA1A), interleukins α and β, dopamine D2 receptor (DA D2) and brain derived neurotrophic factor (BDNF) has been implicated to modulate the extent of injury [12, 17–19], regulating the cascading neurochemical response to the sudden impact or trauma [12,17, 20–27], altering the natural recovery pathways [12–13, 28–38], adversely affecting the cognitive recovery processes [32, 39–46] and behavioral functions [17, 46–52]. BDNF has been implicated in many of these repair processes. It is an abundantly available neurotrophin in the brain that is activity dependent [53–55] with a widespread distribution in the cerebral cortex, hippocampus, basal forebrain, striatum and septum areas [56].

BDNF is also known to play a key role in the survival, differentiation, synaptic plasticity and outgrowth of peripheral and central neurons throughout adulthood [57–60]. Missense mutations within this gene are also known to influence both axonal and dendritic morphology where the ocular dominance column development [61–62] and initial dendritic outgrowth are altered [63–64]. While there are over 1768 missense mutations reported in *BDNF* [65], only two are known to influence the expression level of BDNF, rs6265 (c.196G>A, p.V66M, NM_001143814.1) [58, 66] and a dinucleotide GT microsatellite repeat designated as BDNF-linked complex polymorphic region (BDNF-LCPR) located at the 5’ UTR [58]. The rs6265 variant has been reported to affect the regulated secretion, neural activation, and neuroplastic effect of BDNF as well as neurocognitive functions in humans [29, 66–67]. It has been associated with memory and learning [29, 68–74] and as well as with aspects of executive functioning [66, 75–80], including response inhibition [75], decision making [77–78], task-switching [79], attention shifting and sequencing [80]. Meanwhile, the BDNF-LCPR variants on the other hand have been associated with an increased risk for bipolar disorder [81]. The focus of our study, however, was limited to the broad concepts of BDNF-specific phenotype-modulated structural alteration influencing neuro-regenesis (repair and adaptive synaptic organization) [12, 51] and neurogenesis (active production of neurons, astrocytes, glia and other neural lineages) [12, 52] and its relationship with neurocognition.

Six missense mutations in BDNF [82–84], namely the rs6265, rs1048218, rs1048220, rs1048221, rs8192466 and rs139352447 were selected. The rs6265 variant has been well studied for its involvement in modulating recovery from brain injury but has yet to be investigated in the Malaysian population. The rs1048220 and rs1048221 are within the crucial protease cleavage site for proBDNF and are reported to impair proBDNF cleavage; and rs1048220 and rs104218 have been associated with Alzheimer’s disease [85–89]. However, none of these missense mutations have been explored in brain injury with the exception of rs6265 (BDNF Val66Met) [18, 20, 32, 40, 46, 58, 66, 75]. Hence, the objective of our study was to assess the effects and association of variations within *BDNF* in relation to neurocognitive performance among patients with mTBI.

## Materials and Methods

A total of 61 patients with mTBI who presented to the Emergency Department of University Malaya Medical Center, Kuala Lumpur between April 1^st^, 2013 and August 31^st^, 2014 were recruited prospectively. We defined mTBI as an acute head injury, consisting of non-penetrating head trauma resulting in one or more of the following: confusion/disorientation; loss of consciousness (LOC) less than 30 minutes; posttraumatic amnesia (PTA—less than 24 hours in duration); transient focal neurological signs or seizures; and Glasgow Coma Scale of 13 to 15 upon acute clinical evaluation. These patients were assessed with baseline computed tomography (CT) scans of the brain in the emergency department using a Siemens Somatom Sensation 16 CT scanner (Siemens AG, Berlin, Germany). A neuroradiologist (NR) and a neurosurgeon (VN) who were blinded to the clinical diagnosis independently evaluated the images for each patient. Patients who met the study criteria were admitted to the observation ward for 24 hours. Informed consent was obtained upon explaining the objectives of the study and as well as the research protocols/procedures as per the approved guidelines of our local Ethics Committee for the study (UM/EC/949.15). Thirteen patients were later dropped from this study as some refused screening of their genetic profiles, while others were later lost to follow-up, leaving the final sample of 48 patients with their DNA analyzed for genotyping.

### Genotyping

**Fig 1:**
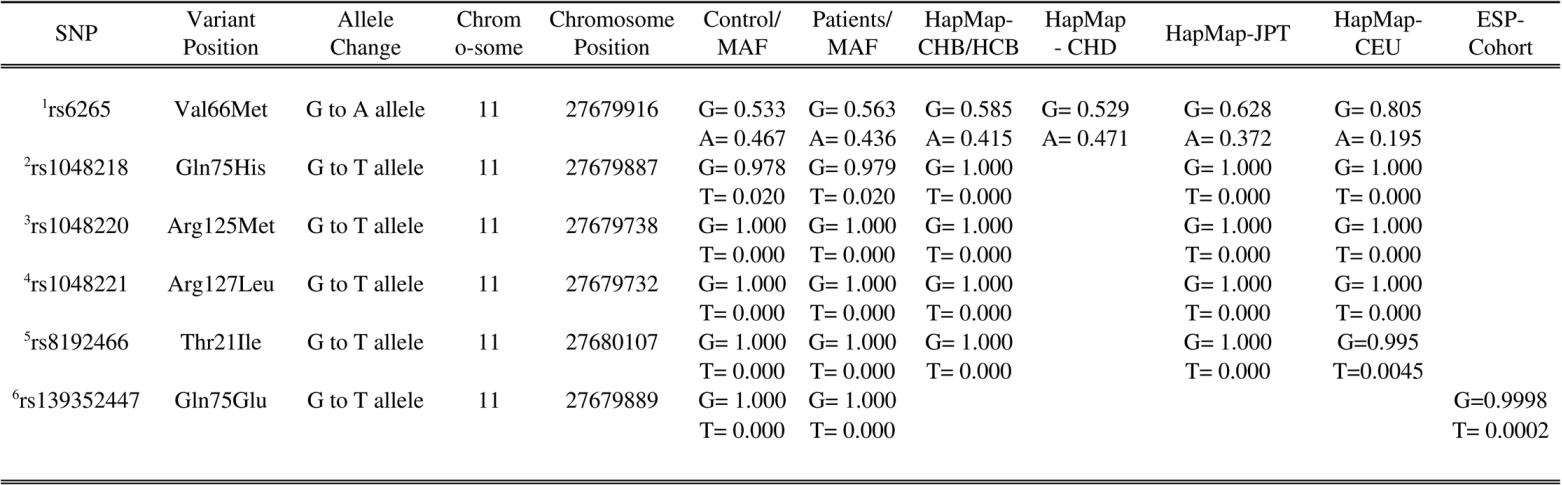
List of BDNF SNPs studied, chromosome position, minor allele frequencies and genotyping quality control values of study healthy subjects, patients with mTBI and comparative haplotype groups. Abbreviation: MAF, Minor Allele Frequency; HapMap, Haplotype Map; CHB/HCB, Han Chinese of Beijing; CHD, Han Chinese of Denver, JPT, Japanese of Tokyo; CEU,Northern and Western European Ancestry, Utah; ESP-Cohort, Exome Sequencing Project Cohort. ^1^ Reference minor allele frequency as reported in http://www.ncbi.nlm.nih.gov/projects/SNP/snp_ref.cgi?rs=6265. ^2^ Reference minor allele frequency as reported in http://www.ncbi.nlm.nih.gov/projects/SNP/snp_ref.cgi?rs=1048218. ^3^ Reference minor allele frequency as reported in http://www.ncbi.nlm.nih.gov/projects/SNP/snp_ref.cgi?rs=1048220. ^4^ Reference minor allele frequency as reported in http://www.ncbi.nlm.nih.gov/projects/SNP/snp_ref.cgi?rs=1048221. ^5^ Reference minor allele frequency as reported in http://www.ncbi.nlm.nih.gov/projects/SNP/snp_ref.cgi?rs=8192466. ^6^ Reference minor allele frequency as reported in http://www.ncbi.nlm.nih.gov/projects/SNP/snp_ref.cgi?rs=139352447.

**Fig 2:**
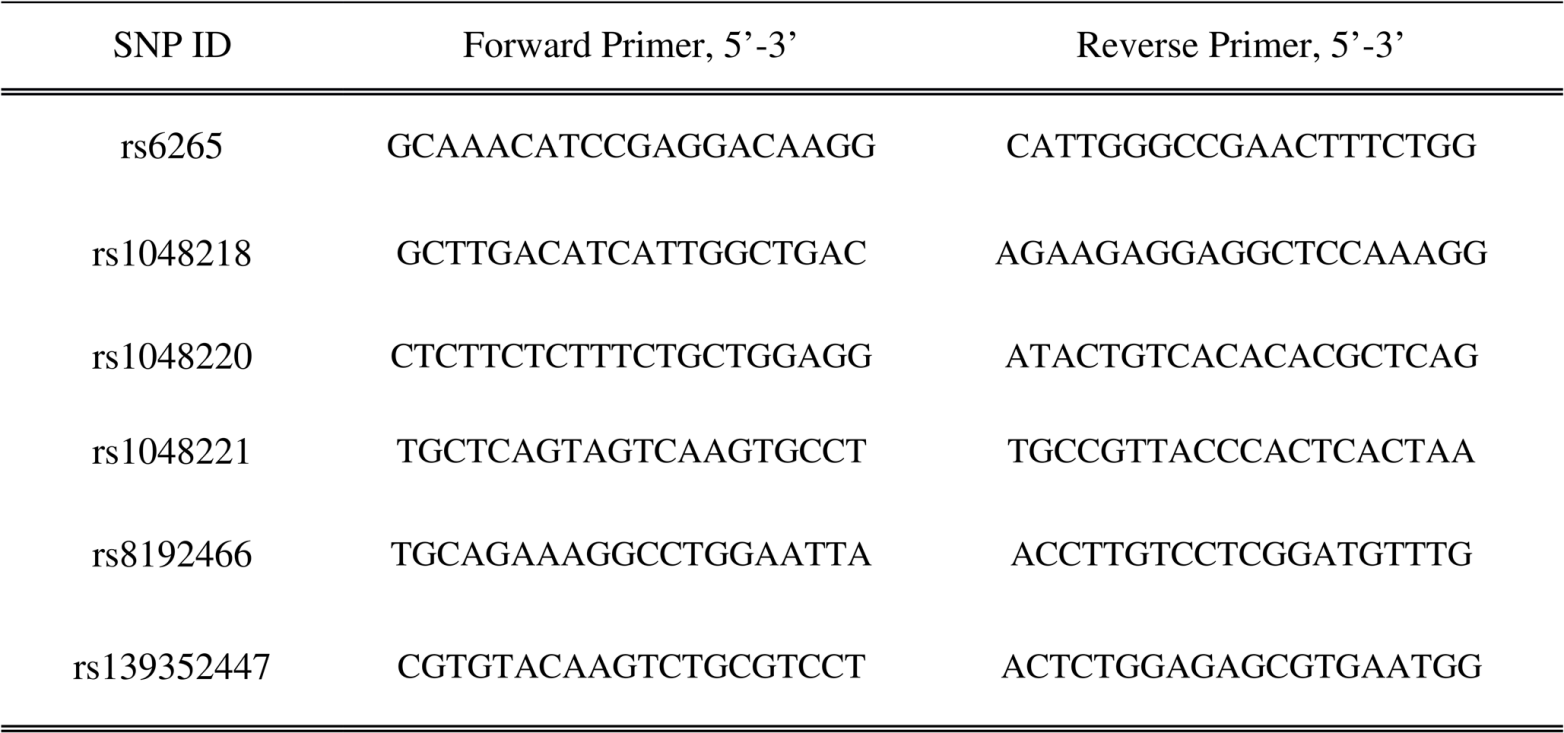
The primer sequences of 6 SNPs of BDNF studied.

DNA was obtained from leukocytes using the phenol-chloroform extraction method [90]. Details of the six SNPs that were examined in this study are in Fig 1. The SNPs were genotyped using Taqman® allelic discrimination assays and genotyping was carried out on a 7500 Fast Real-Time PCR machine (Applied Biosystems) using standard protocols as recommended by the manufacturer. Genotypes were confirmed by polymerase chain reaction (PCR) and Sanger sequencing in a random subset of individuals to determine the error rate for each of the Taqman SNP assays (see Fig 2 for the primer sequences).

## Neurocognitive assessment

**Fig 3:**
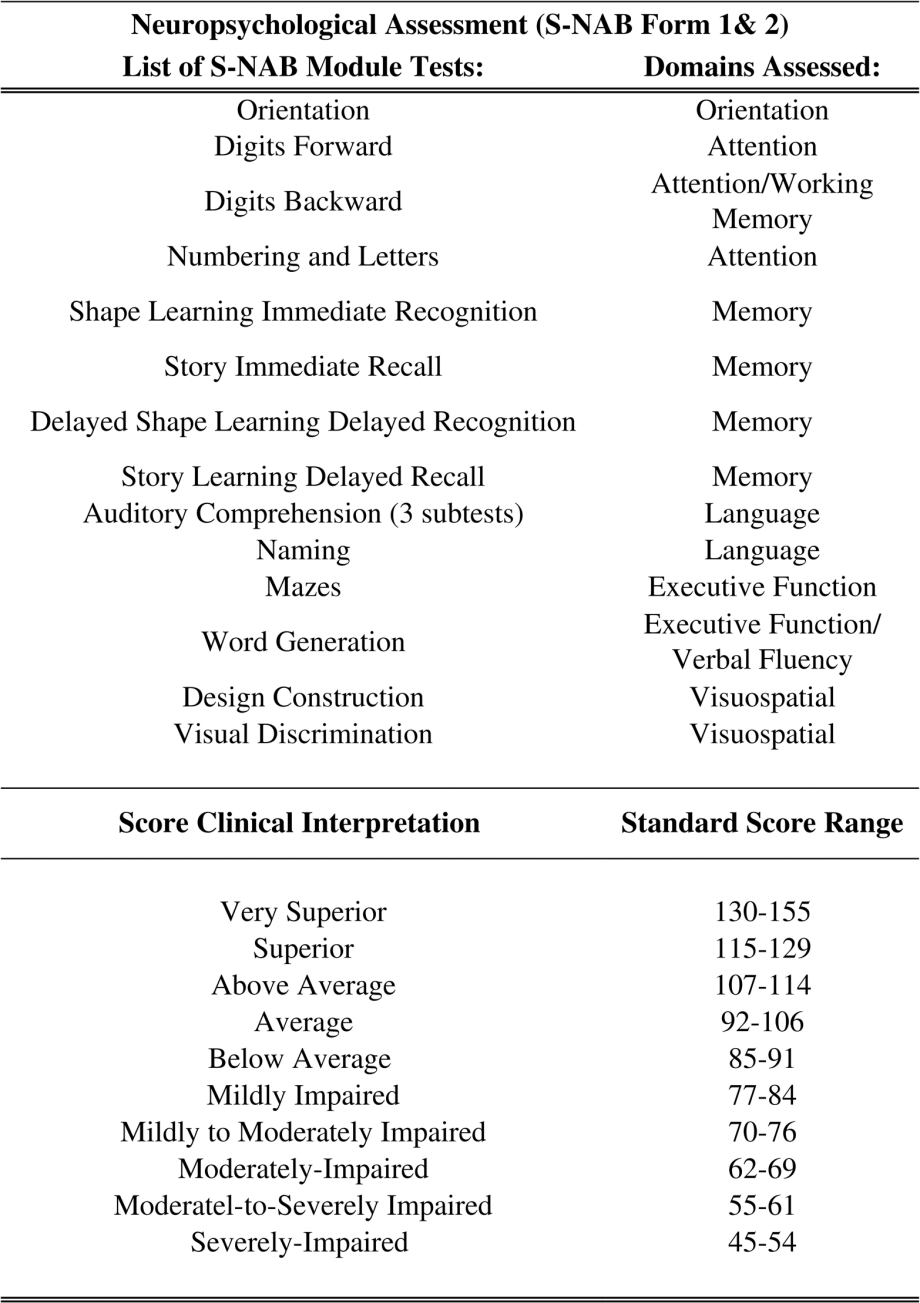
List S-NAB module subtests and areas of cognitive domains assessed, standard score range of individual domains in S-NAB, and clinical interpretation of the scores.

The screening module of Neuropsychological Assessment Battery (S-NAB Form 1) was used to assess the neurocognitive performance of the patients by a clinical neuropsychologist (VV). The assessments were done once the patient had recovered to a GCS score of 15 and was not under any trauma related physical or emotional distress. The S-NAB comprises a comprehensive set of neuropsychological tests (refer to Fig 3), with demographically corrected norms for adults between the ages of 18 to 97 years. Five cognitive domains i.e attention, memory, language, visuospatial and executive functions are evaluated through this battery. This battery consists of 12 individual tests across the five domains aforementioned. A total of 16 *T* scores are then derived, 14 of which contribute toward five separate Screening Index (domain-specific) scores and one Total Screening Index score [91–92]. The S-NAB Form 2 was used to repeat the same subtests in the screening module at 6 months by the same neuropsychologist to assess the neurocognitive performance longitudinally.

### Statistical analysis

All data management and analyses were performed using the SPSS statistical software (Version 22.0). Independent t-test was used to establish the differences in demographic features of the sample, if any, based on their BDNF SNPs and allele status. The mean differences of the standard score (SS) across the time points against the allele carrier status [wildtype G allele (Val homozygotes) vs. minor A allele (Met carriers)] were then analyzed using the paired *t*-test for both categories. The Cohen’s *d* effect size (ES) was also used to measure the magnitude of the differences and a comparison of the ES was done. Repeated measure ANOVA was then performed to delineate statistically significant differences between the groups and their neuropsychological performance across the time point (T_0_ = baseline/ admission vs. T_1_ = 6^th^ month follow-up), The Bonferroni post hoc correction for both multiple comparison and confidence interval adjustment were administered. Any statistically significant (p< 0.05) major effects and interaction were then noted. To assess the association between the allele carrier status and neurocognitive performance, the Spearman correlation coefficient test was also used.

## Results

### Demographic Characteristics

**Fig 4:**
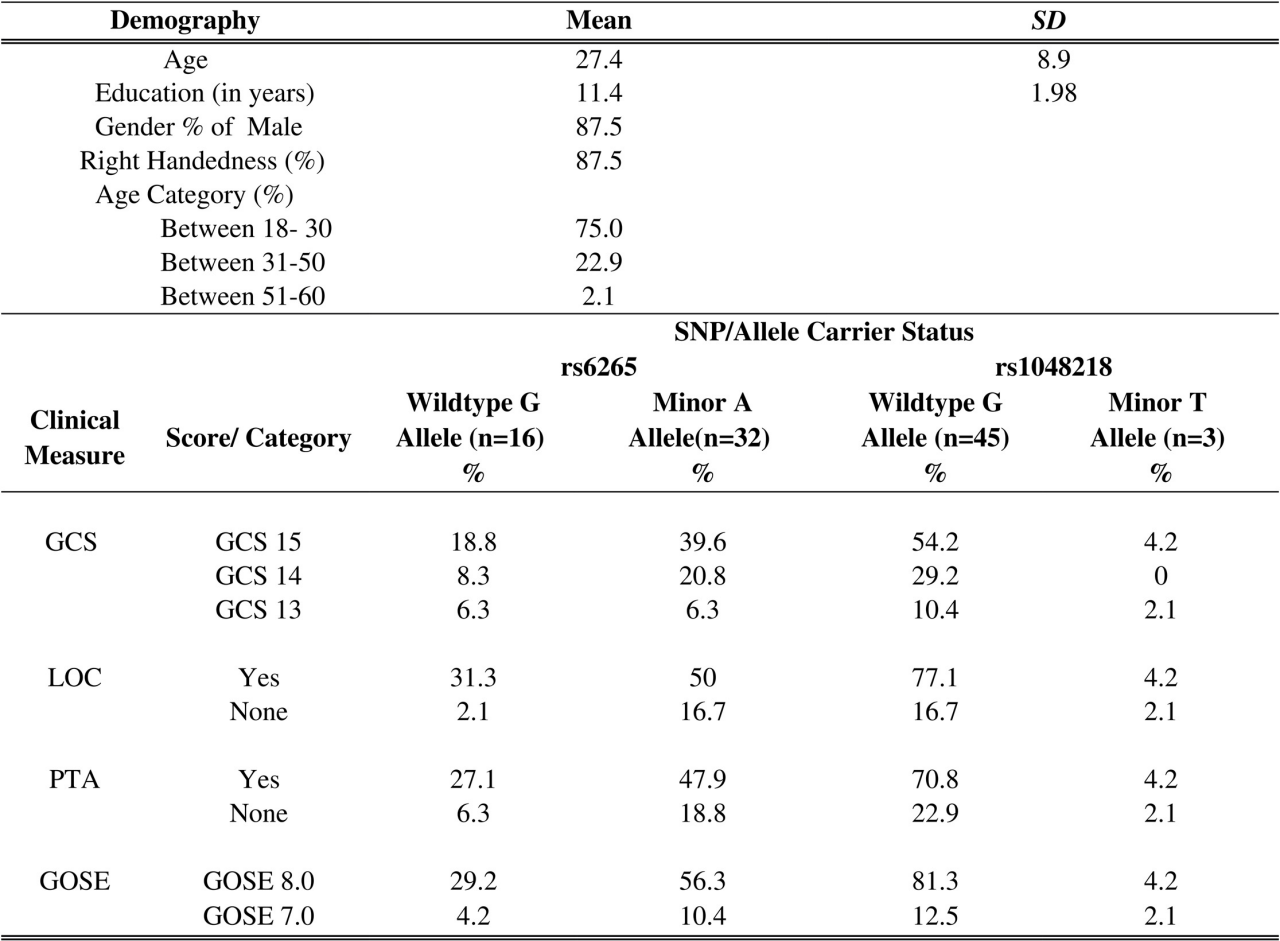
Mean of demographic details and stratified allele status specific clinical measures (in percentage).

The demographic characteristics of the study patients are presented in Fig 4. The study patients were predominantly young males (87.5%), within the age range of 18 to 53 (75.0%) with a mean age of 27.4 (SD 8.9). These patients had an average of 11.4 (SD 2.0) years of formal education. The baseline (T_1_) neuropsychological assessment was conducted after the full GCS recovery of the patients with an average turnaround time of 4.8 hours (SD 7.9) post trauma, while the repeat neuropsychological assessment was done at an average of 6.1 (SD 0.1) months. In order to look at clinically meaningful markers influenced by specific genotypes, we stratified the group according to the SNPs (involving only rs6265 and rs1048218 as the rest of the SNPs were monomorphic as discussed below) and their allele status. No statistically significant differences were observed within the groups except in the presence of LOC (t = -2.026, df = 46, p = 0.049), with a higher incidence among the A minor allele carriers.

### Genetic results and correlation with neurocognitive performance

#### Genotype distribution and minor allele frequency

Six BDNF mutations were examined, of which four (rs1048220, rs1048221, rs8192466, rs139352447) were found to be monomorphic. Only rs6265 and rs1048218 were found to be polymorphic in our population (refer to Fig 1). Both the controls and patients conformed to the Hardy-Weinberg equilibrium for rs6265 and rs1048218. The minor allele frequency for rs6265 was 46.7% in controls compared to the patients (43.6%), but this was not significantly different (p = .380). The high MAF values is comparable to what has been reported previously [93–97] and as annotated for East Asians in the HapMap (41.8%) and 1000genomes (48.8%).

The rs1048218 variant had a low MAF in our population (2% in controls) that is also similar to what has been reported previously and in HapMap and 1000genomes. As the variant was present at a similar frequency in both the patients and controls, no further correlation analysis was performed with this variant.

#### *BDNF* rs6265 vs. neurocognitive performance

**Fig 5:**
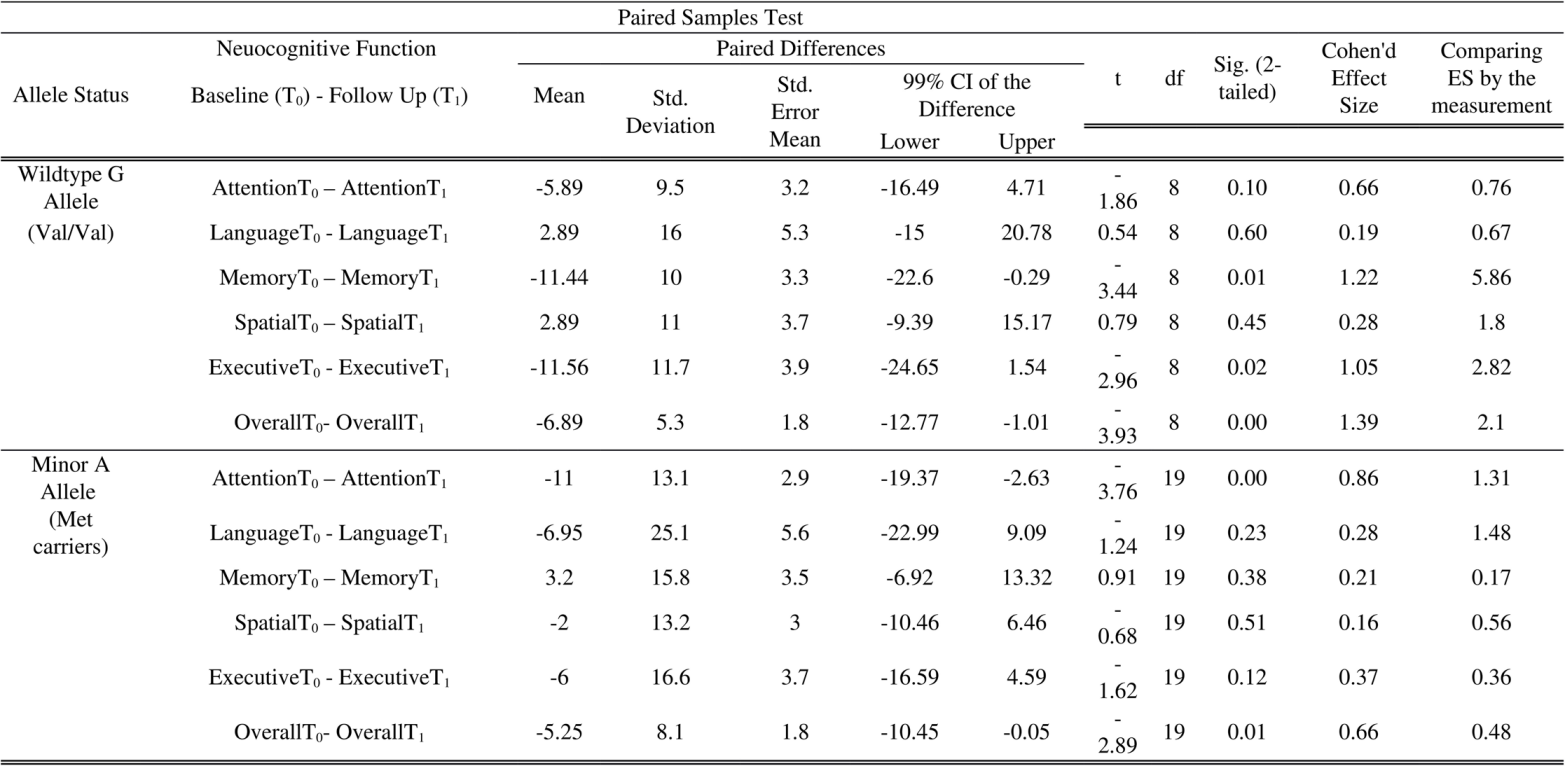
Paired-sample t-test, Cohen’s *d* effect size (ES) calculation, and the comparison of ES by the measurement of the domain specific standard scores (SS) across the time points (admission vs. 6 month follow up) based on the BDNF rs6265 allele status.

Individuals with the A minor allele (corresponding to Met carriers–Met homozygotes/ Met heterozygotes) generally performed more poorly on neuropsychological testing in comparison to those with the wildtype G allele (corresponding to the Val homozygotes) at both time points. Fig 5 presents the significant mean differences as observed among the group wildtype G allele in the domains of memory (*M* = -11.44, SD 10.0, p = .01, *d* = 1.22), executive function (*M* = -11.56, SD = 11.7, p = .02, *d* = 1.05) and overall performance (*M* = -6.89 SD = 5.3, p = .00, *d* = 1.39), while the group with the minor A allele showed significant mean differences in the domains of attention (*M* = -11.0, SD = 13.1, p = .00, *d* = .86) and overall cognitive performance (*M* = -5.25, SD = 8.1, p = .01, *d* = .66).

Further comparison of the effect size by the measurement (i.e. domain-specific SS) demonstrated that the patients with wildtype G allele were 5.86 times more likely to perform better in the domains of attention, 1.8 times in memory, 2.82 times in executive function and 2.1 times higher in overall cognition (total index score) in comparison to the A minor allele over time. Individuals with the minor allele showed considerably lower scores at admission and remained impaired in most domains across the time points, although delayed signs of recovery were noted to be significant in the domains attention and overall cognition.

**Fig 6:**
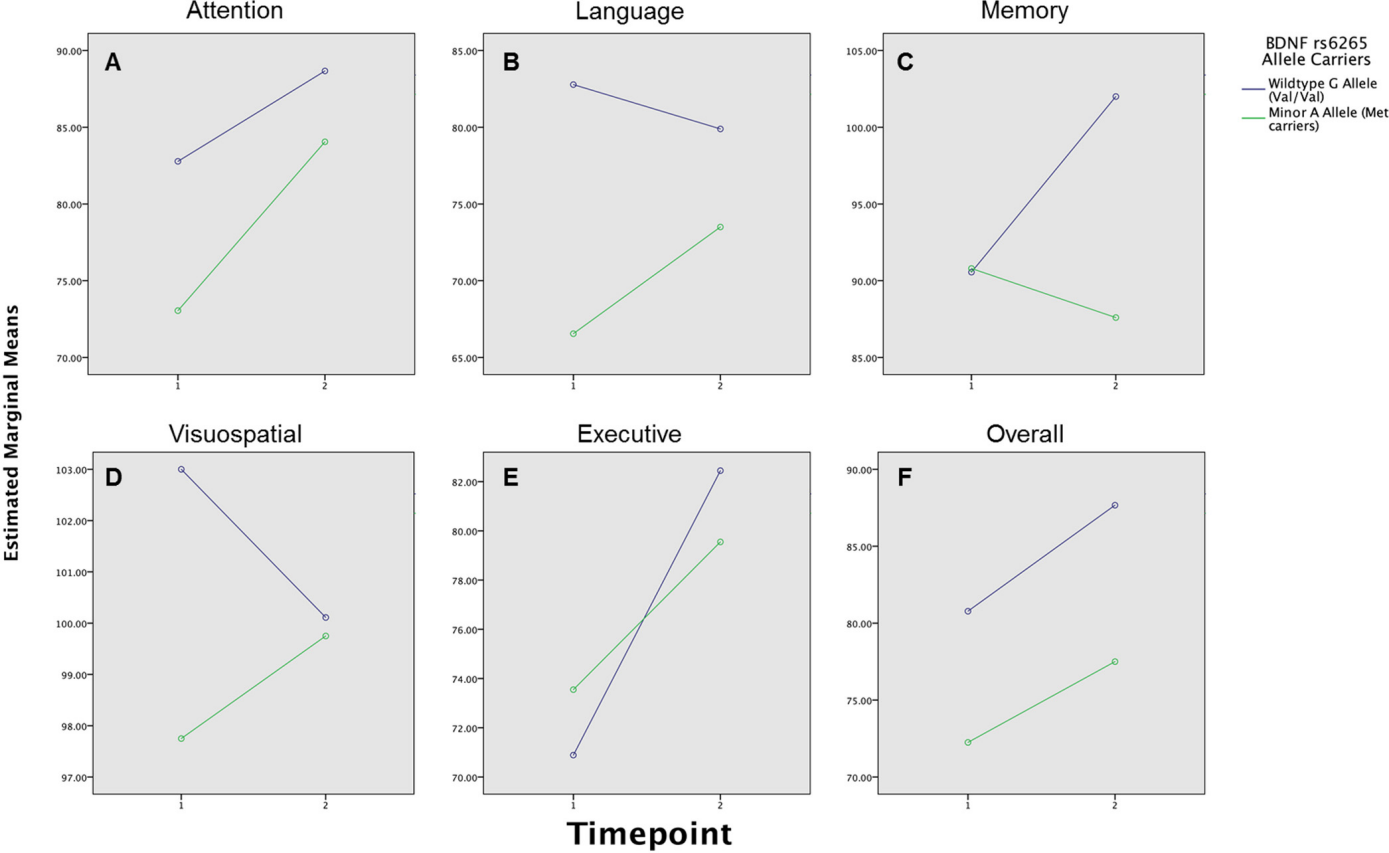
Estimated marginal mean of patients with mTBI, stratified according to their genetic allele status and their domain specific neuropsychological test standard scores across timepoints. (A) Non-significant changes in the attention domain standard score (SS) overtime, with Met carrier performing poorly in the acute stage. (B) Non-significant changes in the language domain standard score (SS) overtime, with the Met carriers, performing poorly acutely, and remaining so overtime. (C) Significant interaction between the allele carrier status and change in memory SS over time with the Met allele carriers showing signs of deterioration at 6 months post trauma. (D) Non- significant changes in the visuospatial SS, intact in both allele groups. (E) Non-significant interactions between allele carrier status and neurocognitive performance within the domains of executive function, with the SS recovery rate being slower in the Met allele carriers. (F) Non-significant changes in the overall index SS, with the Met allele carriers remaining impaired over time.

ANOVA tests revealed that the different time points (T_1_ = admission and T_2_ = 6 month follow-up) produced a significant main effect on neuropsychological *SS* [F (6,22) = 5.786, p < 0.001, η_p_^2^ = .616], which was largely influenced by allele status [F(6,22) = 1.997, p = 0.110, η_p_^2^ = .353] based on the η_p_^2^ value (Eta-squared effect size: 0.02 = small, 0.13 = moderate and 0.3 = large). Some interactions were seen in the neurocognitive domains of attention [F (1,27) = 1.103, p = 0.303], language, F (1,27) = 1.159, p = 0.291, visuospatial, F (1,27) = 0.935, p = 0.342 and executive function [F (1,27) = 0.820, p = 0.373] [as seen in estimated marginal means (EMM) plot in Fig 6A, 6B, 6D and 6E]. However, only memory [Fig 6C] had a statistically significant interaction with the allele status [F (1,27) = 6.476, p = 0.02]. The overall performance showed no interaction [F (1,27) = 0.305, p = 0.585] [see Fig 6F] across the time points and allele status.

No statistically significant associations were observed across the neurocognitive domains and specific allele status with the exception of the memory SS score at 6 months (r = -0.412, *p* = 0.05). The memory scores of patients with the A allele were observed to be significantly lower at six months follow-up.

## Discussion

We explored the prevalence and possible association of six *BDNF* mutations with specific neurocognitive functions in patients with mTBI over time. We observed a possible protective effect of the G allele in rs6265, with better performance in the domains of attention, executive function, memory, and overall cognition among patients with mTBI. The “finer” performance by those patients with the wildtype G allele in both neurocognitive and neurobehavioral measures, have been consistently reported by other studies involving other CNS pathologies as well [32, 88, 98–99].

For example, McAllister et al (2012) demonstrated that patients with mTBI reported better performance on measures of processing speed longitudinally among the G allele homozygotes as opposed to those who were homozygote for the A allele [32]. Similarly, Chao, Kao and Porton (2008) reported that the age of schizophrenia onset in a group of non-related African American patients (n = 42) was significantly later in G allele homozygotes [88]. Moreover, Perkovic et al (2014) showed that patients with schizophrenia who were G allele homozygotes were better responders to pharmacotherapeutic treatment and demonstrated significant improvement of clinical symptoms, including lesser conceptual disorganization and delusions [99].

While some studies have reported increased neurocognitive vulnerability in G allele homozygotes [69, 100–102], we believe that the superior neurocognitive performance we observed in our study may be due to the possibility that the G homozygous allele is associated with increased activity dependent secretion of BDNF, increased synaptic plasticity, and better hippocampus dependent memory and cognitive performance [99]. These mechanistic processes have been well explicated in the works of Egan et al (2003) and Kauppi et al (2013) [29, 72]. The divergent influence of haplotype specific variants cannot be overlooked as well.

On the other hand, patients with the A allele in our study revealed mostly non-significant trends of impaired neurocognitive performance with some interactions seen across the time points in the domains of executive function and overall cognition. Additionally, the memory domain saw statistically significant interaction over time and was also negatively associated with allele status, with evidence of regressing memory function at 6 months among the patients with the G allele. Kauppi et al (2013) noted that this preferential effect on memory is in line with the role of BDNF in the molecular processes underlying memory acquisition. Mechanistically, hippocampal or para-hippocampal BDNF is secreted during neural activation [72]. The increased postsynaptic level of BDNF is known to influence the formation of new synapses in the late phase long term potentiation (LTP), a process that is crucial for the acquisition and storage of long term memories. A successful completion of this crucial molecular process is however impeded when the activity dependent secretion and intracellular trafficking of BDNF is reduced in the protein [29, 72].

Taken together, findings of the current study are the following: (1) only two non-synonymous alterations of the amino acid were present in our study population (rs6265/Val66Met and rs 1048218) and the rest of the remaining variants were monomorphic in nature; (2) mTBI patients with BDNF rs6265 Val homozygous allele showed significant differences in their neurocognitive performance and were more likely to perform better than the Met carriers in the domains of attention, memory, executive function and overall performance, both acutely and over time; (3) the Met allele carriers of BDNF rs6265 had considerably low standard scores in most neurocognitive domains observed longitudinally; (4) there was a significant main effect of the time points, and the influence of specific allele status on neurocognitive performance observed; and (5) longitudinal change in memory performance with evidence of deteriorating performance among the A minor allele group (Met carriers) was observed. Strengths of the study include a relatively well characterized homogeneous group of patients in terms of injury type or severity, a short time frame from the time of injury to neurocognitive testing, detection of early neuropsychological deficits in the acute stage, and a consistent reassessment interval at 6 months post trauma. However, there are certain limitations in our study that are worth noting. First, the method of dichotomizing the patients’ allele carrier status category (wildtype G allele vs. A minor allele, both the homozygous and heterozygotes) may have unequally diminished the dual allele effect of the A minor allele homozygous vs. heterozygotes (Met/Met vs. Val/Met) on the neurocognitive performance. Additionally, the sample size representing each arms of the rs6265 polymorphism was rather small and should be increased in future longitudinal studies.

## Conclusion

In conclusion, the current study has demonstrated the role of the BDNF rs6265 Val66Met polymorphism in influencing specific neurocognitive outcomes in patients with mTBI. Findings were more detrimentally profound among Met allele carriers. Our results strongly suggest that the role of the Val66Met polymorphism in influencing neurostructural alterations and cognitive and behavioral changes post-mTBI should be further explored. Such investigation in future studies may have significant influence over the ways in which mTBI patients are currently managed and their outcomes predicted.

## References

[1] Coronado VG, Xu L, Basavaraju SV, McGuire LC, Wald MM, Faul MD, et al Surveillance for traumatic brain injury-related deaths: United States, 1997–2007. Atlanta: US Department of Health and Human Services, Centers for Disease Control and Prevention; 2011.

[2] World Health Organization. Global status report on road safety: time for action Geneva, Switzerland,WHO: 2009.

[3] World Health Organization. WHO global status report on road safety 2013: supporting a decade of action Geneva, Switzerland, WHO: 2013.

[4] World Health Organization. Neurological Disorders: Public Health Challenges, Geneva, Switzerland, WHO: 2006.

[5] Crawford FC, Vanderploeg RD, Freeman MJ, Singh S, Waisman M, Michaels L, et al APOE genotype influences acquisition and recall following traumatic brain injury. Neurology 2002; 58(7): 1115–1118. 11940706 10.1212/wnl.58.7.1115

[6] Anderson GD, Temkin NR, Dikmen SS, Diaz-Arrastia R, Machamer JE, Farhrenbruch C et al Haptoglobin phenotype and apolipoprotein E polymorphism: relationship to posttraumatic seizures and neuropsychological functioning after traumatic brain injury. Epilepsy & Behavior 2009; 16(3): 501–506. 19766540 10.1016/j.yebeh.2009.08.025 PMC2783358

[7] Kushner D. Mild traumatic brain injury: toward understanding manifestations and treatment. Arch Internal Med, 1998; 158(15): 1617–1624. 9701095 10.1001/archinte.158.15.1617

[8] Blyth BJ, Bazarian JJ. Traumatic alterations in consciousness: traumatic brain injury. Emerg Med Clin North Am, 2010; 28(3): 571–594. 10.1016/j.emc.2010.03.003 20709244 PMC2923650

[9] Willer B, Leddy JJ. Management of concussion and post-concussion syndrome. Curr Treat Options Neurol 2006; 8(5): 415–426. 16901381 10.1007/s11940-006-0031-9

[10] Marshall S, Bayley M, McCullagh S, Velikonja D, Berrigan L. Clinical practice guidelines for mild traumatic brain injury and persistent symptoms. Can Fam Physician 2012; 58(3): 257–267. 22518895 PMC3303645

[11] Ashman TA, Gordon WA, Cantor JB, Hibbard MR. Neurobehavioral consequences of traumatic brain injury. Mt Sinai J Med 2006; 73(7): 999–1005. 17195886

[12] McAllister TW. Genetic factors modulating outcome after neurotrauma. PM&R 2010; 2(12): S241–S252. 21172686 10.1016/j.pmrj.2010.10.005

[13] Pruthi N, Chandramouli BA, Kuttapa TB Rao SL, Subbaskrishna DK, Abrahama MP, et al Apolipoprotein E Polymorphism and Outcome after Mild to Moderate TBI: A Study of Patient Population in India, Neurol India 2010; 58 (2): 264–269. 10.4103/0028-3886.63810 20508347

[14] Dikmen S, Machamer J, Fann JR, Tenkin NR. Rates of symptom reporting following traumatic brain injury. J Intl Neuropsychol Soc 2010; 16: 401–411. 10.1017/S1355617710000196 20188017

[15] Stulemeijer M, Van der Werf S, Borm GF, Vos PE. Early prediction of favourable recovery 6 months after mild traumatic brain injury. J Neurol, Neurosurg Psychiatry 2008; 79(8): 936–942. 17951281 10.1136/jnnp.2007.131250

[16] Bennett ER, Reuter-Rice K, Laskowitz DT. Genetic Influences in Traumatic Brain Injury In: Laskowitz D, Grant G, Eds. Translational Research in Traumatic Brain Injury. Boca Raton (FL): CRC Press/Taylor and Francis Group; 2016 Chapter 9. Available: http://www.ncbi.nlm.nih.gov/books/NBK326717/26583176

[17] DeKosky ST, Kochanek PM, Clark RSB, Ciallella JR, Dixon CE. Secondary Injury After Head Trauma: Subacute and Long-term Mechanisms. Semin Clin Neuropsychiatry 1998; 3(3): 176–185. 10085205

[18] Michael DB, Byers DM, Irwin LN. Gene expression following traumatic brain injury in humans: analysis by microarray. J Clin Neurosci 2005; 12(3), 284–290. 15851083 10.1016/j.jocn.2004.11.003

[19] Dutcher SA, Michael DB. Gene expression in neurotrauma. Neurol Res 2001; 23(2–3): 203–206. 11320600 10.1179/016164101101198343

[20] Gennarelli TA, Graham DI. Neuropathology In Textbook of traumatic brain injury. Silver JM, McAlister TW, Yudofsky SC, editors. Washington DC, American Psychiatric Publishing; 2005 p. 27–50.

[21] Laurer HL, McIntosh TK. Pharmacologic therapy in traumatic brain injury: update on experimental treatment strategies. Curr Pharm Des 2001; 7(15): 1505–1516. 11562295 10.2174/1381612013397285

[22] Martinez-Lucas P, Moreno-Cuesta J, Garcia-Olmo DC, Sanchez-Sanchez F, Escribano-Martinez J, del Pozo AC, Lizan-Garcia M, et al Relationship between the Arg72Pro polymorphism of p53 and outcome for patients with traumatic brain injury. Intensive Care Med 2005; 31: 1168–1173. 16007417 10.1007/s00134-005-2715-0

[23] Kors EE, Terwindt GM, Vermeulen FL, Fitzsimons RB, Jardine PE, Heywood P, et al Delayed cerebral edema andbfatal coma after minor head trauma: Role of the CACNA1A calcium channel subunit gene and relationship with familial hemiplegic migraine. Ann Neurol 2001; 49: 753–760. 11409427 10.1002/ana.1031

[24] Stam AH, Luijckx GJ, Ginjaar IB, Frants RR, Haan J, Ferrari MD, et al Early seizures and cerebral oedema after trivial head trauma associated with the CACNA1A S218L mutation. J Neurol Neurosurg Psychiatry 2009; 80: 1125–1129. 10.1136/jnnp.2009.177279 19520699

[25] Uzan M, Tanriverdi T, Baykara O, Kafadar A, Sanus GZ, Tureci E, et al Association between interleukin-1beta (IL-1b) gene polymorphism and outcome after head injury: An early report. Acta Neurochir 2005; 147: 715–720. 15891809 10.1007/s00701-005-0529-z

[26] Winter CD, Pringle AK, Clough GF, Church MK. Raised parenchymal interleukin-6 levels correlate with improved outcome after traumatic brain injury. Brain 2004; 127: 315–320. 14645145 10.1093/brain/awh039

[27] Quan N, Herkenham M. Connecting cytokines and brain: A review of current issues. Histol Histopathol 2002; 17: 273–288. 11813877 10.14670/HH-17.273

[28] Hicks RR, Numan S, Dhillon HS, Prasad MR, Seroogy KB. Alterations in BDNF and NT-3 mRNAs in rat hippocampus after experimental brain trauma. Mol Brain Res 1997; 48: 401–406. 9332737 10.1016/s0169-328x(97)00158-7

[29] Egan MF, Kojima M, Callicott JH, Goldberg TE, Kolachana BS, Bertolino A, et al The BDNF val66met polymorphism affects activity-dependent secretion of BDNF and human memory and hippocampal function. Cell 2003; 112(2): 257–69. 12553913 10.1016/s0092-8674(03)00035-7

[30] Poo M. Neurotrophins as synaptic modulators. Nat Rev Neurosci 2001; 2: 24–32. 11253356 10.1038/35049004

[31] Hicks RR, Zhang L, Dhillon HS, Prasad MR, Seroogy KB. Expression of trkB mRNA is altered in rat hippocampus after experimental brain trauma. Mol Brain Res 1998; 59: 264–268. 9729420 10.1016/s0169-328x(98)00158-2

[32] McAllister TW, Tyler AL, Flashman LA, Rhodes CH, McDonald BC, Saykin AJ, et al Polymorphisms in the brain-derived neurotrophic factor gene influence memory and processing speed one month after brain injury. J Neurotrauma 2012; 29(6): 1111–1118. 10.1089/neu.2011.1930 22188054 PMC3325555

[33] Hartman RE, Laurer H, Longhi L, Bales KR, Paul SM, McIntosh TK, et al Apolipoprotein E4 influences amyloid deposition but not cell loss after traumatic brain injury in a mouse model of Alzheimer’s disease. J Neurosci 2002; 22: 10083–10087. 12451108 10.1523/JNEUROSCI.22-23-10083.2002 PMC6758744

[34] Nathoo N, Chetry R, van Dellen JR, Connolly C, Naidoo R. Apolipoprotein E polymorphism and outcome after closed traumatic brain injury: Influence of ethnic and regional differences. J Neurosurg 2003; 98: 302–306. 12593615 10.3171/jns.2003.98.2.0302

[35] Chiang MF, Chang JG, Hu CJ. Association between apolipoprotein E genotype and outcome of traumatic brain injury. Acta Neurochir 2003; 145: 649–654. 14520543 10.1007/s00701-003-0069-3

[36] Alexander S, Kerr ME, Kim Y, Kamboh MI, Beers SR, Conley YP. Apolipoprotein E4 allele presence and functional outcome after severe traumatic brain injury. J Neurotrauma 2007; 24: 790–797. 17518534 10.1089/neu.2006.0133

[37] Kulbatski I, Mothe AJ, Nomura H, Tator CH. Endogenous and exogenous CNS derived stem/progenitor cell approaches for neurotrauma. Curr Drug Targets 2005; 6: 111–126. 15720218 10.2174/1389450053345037

[38] Parasuraman R, Greenwood PM. The apolipoprotein E gene, attention, brain function. Neuropsychology 2002; 16: 254–274. 11949718 10.1037//0894-4105.16.2.254 PMC1350934

[39] Morley KI, Montgomery GW. The genetics of cognitive processes: Candidate genes in humans and animals. Behav Genet 2001; 31: 511–531. 11838530 10.1023/a:1013337209957

[40] Failla MD, Juengst SB, Arenth PM, Wagner AK. Preliminary Associations Between Brain-Derived Neurotrophic Factor, Memory Impairment, Functional Cognition, and Depressive Symptoms Following Severe TBI. Neurorehabil Neural Repair 2015, 1545968315600525 26276123 10.1177/1545968315600525 PMC4752939

[41] Savitz J, Solms M, Ramesar R. The molecular genetics of cognition: Dopamine, COMT and BDNF. Genes Brain Behav 2006; 5: 311–328. 16716201 10.1111/j.1601-183X.2005.00163.x

[42] Kraus MF, Smith GS, Butters M, Donnell AJ, Dixon E, Yilong C, et al Effects of the dopaminergic agent and NMDA receptor antagonist amantadine on cognitive function, cerebral glucose metabolism and D2 receptor availability in chronic traumatic brain injury: a study using positron emission tomography (PET). Brain Injury 2005; 19(7): 471–479. 16134735 10.1080/02699050400025059

[43] Bales J. W., Kline A. E., Wagner A. K., & Dixon C. E. (2010). Targeting dopamine in acute traumatic brain injury. The open drug discovery journal, 2, 119 22308176 10.2174/1877381801002010119 PMC3269831

[44] Yue JK, Winkler EA, McAllister TW, Temkin N, Ferguson A, Lingsma HF, et al 178 COMT Val158Met is Associated With Domain-Specific Cognitive Impairment Following Mild Traumatic Brain Injury. Neurosurgery 2015; 62: 225–225.

[45] Kurowski BG, Backeljauw B, Zang H, Zhang N, Martin LJ, Pilipenko V, et al Influence of Catechol-O-methyltransferase on Executive Functioning Longitudinally After Early Childhood Traumatic Brain Injury: Preliminary Findings. J Head Trauma Rehabil 2015; 10.1097/HTR.0000000000000162 PMC4724555 26394291

[46] Failla MD, Conley YP, Wagner AK. Brain-Derived Neurotrophic Factor (BDNF) in Traumatic Brain Injury–Related Mortality Interrelationships Between Genetics and Acute Systemic and Central Nervous System BDNF Profiles. Neurorehabil Neural Repair 2016; 30(1): 83–93. 10.1177/1545968315586465 25979196 PMC4644728

[47] Ebstein RP, Benjamin J, Belmaker RH. Personality and polymorphisms of genes involved in aminergic neurotransmission. Eur J Pharmacol 2000;410:205–214. 11134670 10.1016/s0014-2999(00)00852-9

[48] Comings DE, Rosenthal RJ, Lesieur HR, Rugle LJ, Muhleman D, Chiu C, et al A study of the dopamine D2 receptor gene in pathological gambling. Pharmacogenet Genomics 1996; 6(3): 223–234. 10.1097/00008571-199606000-00004 8807661

[49] Gogos JA, Morgan M, Luine V, Santha M, Ogawa S, Pfaff D, et al Catechol-O methyltransferase deficient mice exhibit sexually dimorphic changes in catecholamine levels and behavior. Proc Natl Acad Sci 1998; 95: 9991–9996. 9707588 10.1073/pnas.95.17.9991 PMC21449

[50] Wang JC, Hinrichs AL, Stock H, Budde J, Allen R, Bertelsen S, et al Evidence of common and specific genetic effects: Association of the muscarinic acetylcholine receptor M2 (CHRM2) gene with alcohol dependence and major depressive syndrome. Hum Mol Genet 2004; 13: 1903–1911. 15229186 10.1093/hmg/ddh194

[51] Ariza M, Pueyo R, Matarin Mdel M, Junque C, Mataro M, Clemente I, et al Influence of APOE polymorphism on cognitive and behavioural outcome in moderate and severe traumatic brain injury. J Neurol, Neurosurg Psychiatry 2006; 77(10): 1191–1193. 10.1136/jnnp.2005.085167 PMC2077553 16614010

[52] McAllister TW. Genetic factors in traumatic brain injury. Handb Clin Neurol 2014; 128: 723–739. 10.1016/B978-0-444-63521-1.00045-5 25701917

[53] Karpova NN. Role of BDNF epigenetics in activity-dependent neuronal plasticity. Neuropharmacology 2014; 76: 709–718 10.1016/j.neuropharm.2013.04.002 23587647

[54] Cohen‐Cory S, Kidane AH, Shirkey NJ, Marshak S. Brain‐derived neurotrophic factor and the development of structural neuronal connectivity. Dev Neurobiol, 2010; 70(5): 271–288. 10.1002/dneu.20774 20186709 PMC2893579

[55] Korley FK, Diaz-Arrastia R, Wu AH, Yue JK, Manley GT, Sair HI, et al Circulating Brain-Derived Neurotrophic Factor Has Diagnostic and Prognostic Value in Traumatic Brain Injury. J Neurotrauma 2016; 33(2): 215–225. 10.1089/neu.2015.3949 26159676 PMC4722554

[56] Wang H, Zhang Y, Qiao M. Mechanisms of extracellular signal-regulated kinase/cAMP response element-binding protein/brain-derived neurotrophic factor signal transduction pathway in depressive disorder. Neural Regen Res 2013; 8(9): 843 10.3969/j.issn.1673-5374.2013.09.009 25206732 PMC4146087

[57] Huang CC, Liu ME, Chou KH, Yang AC, Hung CC, Hong CJ, et al Effect of BDNF Val66Met polymorphism on regional white matter hyperintensities and cognitive function in elderly males without dementia. Psychoneuroendocrinology 2014; 39: 94–103. 10.1016/j.psyneuen.2013.09.027 24275008

[58] Rostami E, Krueger F, Zoubak S, Dal-Monte O, Raymont V, Pardini M, et al BDNF polymorphism predicts general intelligence after penetrating traumatic brain injury. PloS One 2011; 6(11): e27389 10.1371/journal.pone.0027389 22087305 PMC3210804

[59] Binder DK, Scharfman HE. Mini review. Growth Factors 2004; 22(3): 123–131. 15518235 10.1080/08977190410001723308 PMC2504526

[60] Hong CJ, Liou YJ, Tsai SJ. Effects of BDNF polymorphisms on brain function and behavior in health and disease. Brain Res Bul 2011; 86(5): 287–297. 10.1016/j.brainresbull.2011.08.019 21924328

[61] Cabelli RJ, Shelton DL, Segal RA, Shatz CJ. Blockade of endogenous ligands of trkB inhibits formation of ocular dominance columns. Neuron, 1997; 19(1): 63–76. 9247264 10.1016/s0896-6273(00)80348-7

[62] Horch HW, Kruttgen A, Portbury SD, Katz, LC. Destabilization of cortical dendrites and spines by BDNF. Neuron 1999; 23(2): 353–364. 10399940 10.1016/s0896-6273(00)80785-0

[63] McAllister AK, Katz LC, Lo DC. Neurotrophin regulation of cortical dendritic growth requires activity. Neuron 1996; 17(6): 1057–1064. 8982155 10.1016/s0896-6273(00)80239-1

[64] McAllister AK, Katz LC, Lo DC. Neurotrophins and synaptic plasticity. Ann Rev Neuroscience 1999; 22(1): 295–318. 10.1146/annurev.neuro.22.1.295 10202541

[65] 1000 Genomes Project Consortium. An integrated map of genetic variation from 1,092 human genomes. Nature 2012; 491(7422): 56–65. 10.1038/nature11632 23128226 PMC3498066

[66] Barbey AK, Colom R, Paul E, Forbes C, Krueger F, Goldman D, et al Preservation of general intelligence following traumatic brain injury: contributions of the Met66 brain-derived neurotrophic factor. PloS One 2014; 9(2): e88733 10.1371/journal.pone.0088733 24586380 PMC3935849

[67] Tost H, Alam T, Geramita M, Rebsch C, Kolachana B, Dickinson D, et al Effects of the BDNF Val66Met polymorphism on white matter microstructure in healthy adults. Neuropsychopharmacology 2013; 38(3): 525–532. 10.1038/npp.2012.214 23132269 PMC3547204

[68] Hariri AR, Goldberg TE, Mattay VS, Kolachana BS, Callicott JH, Egan MF, et al Brain-derived neurotrophic factor val66met polymorphism affects human memory-related hippocampal activity and predicts memory performance. J Neurosci 2003; 23(17): 6690–6694. 12890761 10.1523/JNEUROSCI.23-17-06690.2003 PMC6740735

[69] Bekinschtein P, Cammarota M, Izquierdo I, Medina JH. Reviews: BDNF and memory formation and storage. Neuroscientist 2008; 14(2): 147–156. 17911219 10.1177/1073858407305850

[70] Miyajima F, Ollier W, Mayes A, Jackson A, Thacker N, Rabbitt, et al Brain‐derived neurotrophic factor polymorphism Val66Met influences cognitive abilities in the elderly. Genes, Brain and Behav 2008; 7(4): 411–417. 10.1111/j.1601-183X.2007.00363.x 17973920

[71] Voineskos AN, Lerch JP, Felsky D, Shaikh S, Rajji TK, Miranda D et al The brain-derived neurotrophic factor Val66Met polymorphism and prediction of neural risk for Alzheimer disease. Arch Gen Psychiatry, 2011; 68(2): 198–206. 10.1001/archgenpsychiatry.2010.194 21300947

[72] Kauppi K, Nilsson LG, Adolfsson R, Lundquist A, Eriksson E, Nyberg L. Decreased medial temporal lobe activation in BDNF 66 Met allele carriers during memory encoding. Neuropsychologia 2013; 51(12): 2462–2468. 10.1016/j.neuropsychologia.2012.11.028 23211991

[73] Hashimoto R, Moriguchi Y, Yamashita F, Mori T, Nemoto K, Okada T, et al Dose-dependent effect of the Val66Met polymorphism of the brain-derived neurotrophic factor gene on memory-related hippocampal activity. Neurosci Res 2008; 61(4): 360–367. 10.1016/j.neures.2008.04.003 18501457

[74] Ninan I. Synaptic regulation of affective behaviors; role of BDNF. Neuropharmacology 2014; 76: 684–695. 10.1016/j.neuropharm.2013.04.011 23747574 PMC3825795

[75] Krueger F, Pardini M, Huey ED, Raymont V, Solomon J, Lipsky RH, et al The role of the Met66 brain-derived neurotrophic factor allele in the recovery of executive functioning after combat-related traumatic brain injury. J Neurosci, 2011; 31(2): 598–606. 10.1523/JNEUROSCI.1399-10.2011 21228168 PMC3195417

[76] Beste C, Kolev V, Yordanova J, Domschke K, Falkenstein M, Baune BT, et al The role of the BDNF Val66Met polymorphism for the synchronization of error-specific neural networks. J Neurosci 2010; 30(32): 10727–10733. 10.1523/JNEUROSCI.2493-10.2010 20702703 PMC6634693

[77] Rybakowski JK, Borkowska A, Czerski PM, Skibinska M, Hauser J. Polymorphism of the brain‐derived neurotrophic factor gene and performance on a cognitive prefrontal test in bipolar patients. Bipolar Disord, 2003; 5(6): 468–472. 14636373 10.1046/j.1399-5618.2003.00071.x

[78] Rybakowski JK, Borkowska A, Skibinska M, Hauser J. Illness-specific association of val66met BDNF polymorphism with performance on Wisconsin Card Sorting Test in bipolar mood disorder. Mol Psychiatry, 2006; 11(2): 122–124. 16222333 10.1038/sj.mp.4001765

[79] Gajewski PD, Hengstler JG, Golka K, Falkenstein M, Beste C. The Met-allele of the BDNF Val66Met polymorphism enhances task switching in elderly. Neurobiol Aging, 2011; 32(12): 2327.e7–2327.e19. 10.1016/j.neurobiolaging.2011.06.010 21803453

[80] Getzmann S, Gajewski PD, Hengstler JG, Falkenstein M, Beste C. BDNF Val66Met polymorphism and goal-directed behavior in healthy elderly—evidence from auditory distraction. NeuroImage 2013; 64: 290–298. 10.1016/j.neuroimage.2012.08.079 22963854

[81] Okada T, Hashimoto R, Numakawa T, Iijima Y, Kosuga A, Tatsumi M, et al A complex polymorphic region in the brain-derived neurotrophic factor (BDNF) gene confers susceptibility to bipolar disorder and affects transcriptional activity. Mol Psychiatry 2006; 11(7): 695–703. 16568151 10.1038/sj.mp.4001822

[82] UniProt Consortium. *UniProtKB—P23560 (BDNF_HUMAN)* [online] Available: http://www.uniprot.org/uniprot/P23560#subcellular_location [Accessed 23 October, 2015]

[83] Cunningham F, Amode MR, Barrell D, Beal K, Billis K, Brent S, et al Ensembl. Nucleic Acids Res 2015; 43: D662–D669. 10.1093/nar/gku101 25352552 PMC4383879

[84] Sherry ST, Ward MH, Kholodov M, Baker J, Phan L, Smigielski EM, Sirotkin K. dbSNP: the NCBI database of genetic variation. Nucleic Acids Res 2001; 29(1): 308–11. 11125122 10.1093/nar/29.1.308 PMC29783

[85] Lin WJ, Salton SR. The regulated secretory pathway and human disease: insights from gene variants and single nucleotide polymorphisms. Front Endocrinol, 2013; 4. 10.3389/fendo.2013.00096 PMC3734370 23964269

[86] Huang R, Huang J, Cathcart H, Smith S, Poduslo SE. Genetic variants in brain-derived neurotrophic factor associated with Alzheimer’s disease. J Med Genets 2007; 44(2): e66. 10.1136/jmg.2006.044883 PMC2598055 17293537

[87] Jonsson EG, Edman-Ahlbom B, Sillen A, Gunnar A, Kulle B, Frigessi A, et al Brain-derived neurotrophic factor gene (BDNF) variants and schizophrenia: an association study. Prog Neuropsychopharmacol Biol Psychiatry 2006; 30(5): 924–933. 16581172 10.1016/j.pnpbp.2006.02.008

[88] Chao HM, Kao HT, Porton B. BDNF Val66Met variant and age of onset in schizophrenia. Am J Med Genet B Neuropsychiatr Genet 2008; 147(4): 505–506. 10.1002/ajmg.b.30619 PMC2396782 17894414

[89] Weese‐Mayer DE, Bolk S, Silvestri JM, Chakravarti A. Idiopathic congenital central hypoventilation syndrome: Evaluation of brain‐derived neurotrophic factor genomic DNA sequence variation. Am J Med Genet A, (2002; 107(4): 306–310. 10.1002/ajmg.10133 11840487

[90] Miller SA, Dykes DD, Polesky HFRN. A simple salting out procedure for extracting DNA from human nucleated cells. Nucleic Acids Res 1988; 16(3): 1215 3344216 10.1093/nar/16.3.1215 PMC334765

[91] Veeramuthu V, Narayanan NV, Tan LK, Delano-Wood L, Chinna K, Bondi MW, et al Diffusion Tensor Imaging Parameters in Mild Traumatic Brain Injury and Its Correlation with Early Neuropsychological Impairment: A Longitudinal Study. J Neurotrauma 2015; 32(19): 1497–1509. 10.1089/neu.2014.3750 25952562 PMC4589266

[92] Stern RA, White T. Neuropsychological Assessment Battery. Lutz, FL: Psychological Assessment Resources 2003.

[93] Mohajeri MH, Giese KP. Two selected models of missense mutations in mice for the study of learning behaviour. Brain Res Bul 2012; 88(5): 429–433. 10.1016/j.brainresbull.2011.12.003 22214603

[94] Pezawas L, Verchinski BA, Mattay VS, Callicott JH, Kolachana BS, Straub RE, et al The brain-derived neurotrophic factor val66met polymorphism and variation in human cortical morphology. J Neurosci 2004; 24(45): 10099–10102. 15537879 10.1523/JNEUROSCI.2680-04.2004 PMC6730170

[95] Pivac N, Kim B, Nedic G, Ho-Joo Y, Kozaric-Kovacic D, Pyo Hong J, et al Ethnic differences in brain derived neurotrophic factor Val66Met polymorphism in Croatian and Korean healthy participants. Croat Med J 2009; 50(1): 49–54. 19260143 10.3325/cmj.2009.50.43 PMC2657559

[96] Shimizu E, Hashimoto K, Iyo M. Ethnic difference of the BDNF 196G/A (val66met) polymorphism frequencies: the possibility to explain ethnic mental traits. Am J Med Genet B Neuropsychiatr Genet 2004; 126(1): 122–123. 10.1002/ajmg.b.20118 15048661

[97] Petryshen TL, Sabeti PC, Aldinger KA, Fry B, Fan JB, Schaffner SF, et al Population genetic study of the brain-derived neurotrophic factor (BDNF) gene. Mol Psychiatry 2010; 15(8): 810–815. 10.1038/mp.2009.24 19255578 PMC2888876

[98] Kang JI, Namkoong K, Ha RY, Jhung K, Kim YT, Kim S. J. Influence of BDNF and COMT polymorphisms on emotional decision making. Neuropharmacology 2010; 58(7): 1109–1113. 10.1016/j.neuropharm.2010.02.001 20153759

[99] Perkovic MN, Erjavec GN, Zivkovic M, Sagud M, Uzun S, Mihaljevic-Peles A, et al Association between the brain-derived neurotrophic factor Val66Met polymorphism and therapeutic response to olanzapine in schizophrenia patients. Psychopharmacology 2014; 231(18): 3757–3764. 10.1007/s00213-014-3515-4 24595507

[100] Dennis NA, Cabeza R, Need AC, Waters‐Metenier S, Goldstein DB, LaBar KS. Brain‐derived neurotrophic factor val66met polymorphism and hippocampal activation during episodic encoding and retrieval tasks. Hippocampus 2011; 21(9): 980–989. 10.1002/hipo.20809 20865733 PMC3010486

[101] van Wingen G, Rijpkema M, Franke B, van Eijndhoven P, Tendolkar I, Verkes RJ, et al The brain-derived neurotrophic factor Val66Met polymorphism affects memory formation and retrieval of biologically salient stimuli. Neuroimage, 2010; 50(3), 1212–1218. 10.1016/j.neuroimage.2010.01.058 20097294

[102] Harris SE, Fox H, Wright AF, Hayward C, Starr JM, Whalley LJ, et al The brain-derived neurotrophic factor Val66Met polymorphism is associated with age-related change in reasoning skills. Mol Psychiatry 2006; 11(5): 505–513. 16446742 10.1038/sj.mp.4001799

